# Locking plate constructs in subtrochanteric fixation: a biomechanical comparison of LCP screws and AO-nuts

**DOI:** 10.1016/j.jcot.2020.12.026

**Published:** 2020-12-31

**Authors:** T.P.A. Baltes, A.J. van der Veen, L. Blankevoort, J.C.E. Donders, P. Kloen

**Affiliations:** a Department of Orthopaedic Surgery, Amsterdam UMC, University of Amsterdam, Amsterdam Movement Sciences, Amsterdam, the Netherlands; b Department of Physics and Medical Technology, Amsterdam UMC, Vrije Universiteit Amsterdam, Amsterdam Movement Sciences, Amsterdam, the Netherlands

**Keywords:** Fixed-angle construct, Condylar blade-plate, AO-nut, Fracture, Non-union

## Abstract

**Objectives:**

Various studies have reported the use of the 95-degree condylar blade plate in the treatment of a subtrochanteric fracture or non-union. However, the holding power of standard screws in the metaphyseal and diaphyseal area is often diminished due to osteopenia. The alternative in this area is the use of locking plates, Schühlis or AO-nuts. With the latter two, non-locking screws in the blade plate can be converted to a fixed angle fixation. The objective of this study was to compare the stiffness and strength of the AO-nut augmented 95-degree condylar blade plate construct with that of a locking plate construct. In addition, a clinical series of eight patients treated with the AO-nut augmented 95-degree condylar blade plate construct is presented.

**Methods:**

Single screw-plate constructs of a 5.0 mm locking screw/locking compression plate (LCP) and a 4.5 mm non-locking screw/4.5 mm dynamic compression plate (DCP), converted to a fixed-angle screw construct using AO-nuts, were tested by cantilever bending. During loading, force and displacement were recorded, from which the bending stiffness (N/mm) and the yield strength (N) were determined. Secondarily, all patients that underwent surgical treatment for subtrochanteric fracture, malunion or non-union by the senior author using this technique, underwent chart review.

**Results:**

The stiffness of the locking screws was about four times higher compared to the AO-nut augmented construct. The yield strength was 2.3 times higher for the locking screw construct. In none of the eight patients treated with the fixed-angle blade plate, failure of the AO-nut augmented construct occurred.

**Conclusions:**

Although the stiffness and strength of the AO-nut augmented construct is less than of the locking screw, excellent clinical outcomes can be achieved utilizing this construct.

## Introduction

Subtrochanteric femur fractures constitute 7–34% of all proximal femur fractures.^1^ When treated with contemporary methods of fixation, most fractures heal well. However, a subset of patients develops a non-union or malunion. Revision fixation in the subtrochanteric region can represent a significant challenge, as they often present with large bone voids, scarring, osteopenia, failed hardware and deformity.

Various options have been described in the treatment of subtrochanteric non-unions, including revision to larger intramedullary implants, plate fixation or hip arthroplasty in older patients.^2^ Others have also recognized the 95-degree condylar blade plate as the workhorse in a subtrochanteric non-union, as it is an inexpensive plate and a reliable surgical technique.^3^^,^^4^ In addition, the blade plate allows for correction in multiple planes (e.g. malrotation, flexion/extension and valgus/varus deformities) while only creating a small footprint, making it the ideal implant for subtrochanteric revision surgery.^4^

**Fig. 1:**
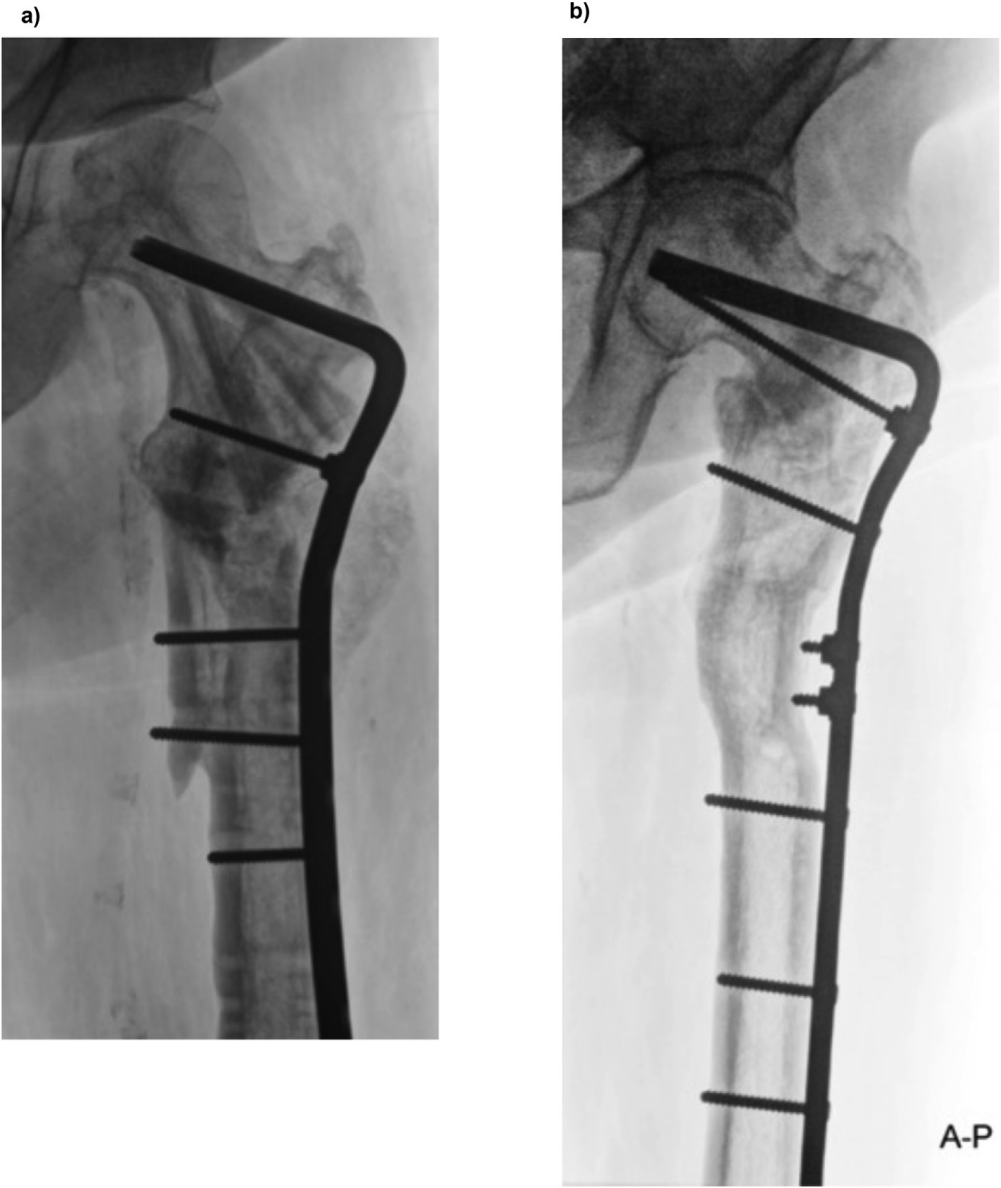
Using the AO-nut a fixed-angle construct can be created; (A) Using the AO-nut a fixed-angle construct is created to substitute the missing lateral cortex of the femur (B) The most cephalad AO-nut is used as described under (A). The two distal AO-nuts are used to create two short fixed-angle screws that push the osteotomized trapezoid wedge medially, thus creating an indirect lengthening of the femur.

Although we commonly use locking plates in our daily practice, catastrophic hardware failure of the proximal femoral locking plate as reported in earlier series has steered us away from its use.^5^^,^^6^ The causes for catastrophic failure are still unclear, but it may be because the fixation with a “too rigid” locking device is suboptimal. Also, we think that the small footprint of the blade plate in the often compromised bone stock of the femoral neck and head is superior than multiple locking screws. The increased holding power of hybrid screw fixation (a mixture of locking and non-locking screws) in the osteopenic/porotic shaft would on the other side optimize fixation of the side plate. The blade plate can be modified into such a hybrid technique whereby the non-locking blade plate design is converted into a construct with fixed angle screw fixation using AO-nuts. (Fig. 1). We think this combines the benefits of compression with a better tactile sense of final fixation, low costs, and limited additional bone loss. In areas of bone loss where the plate does not have appositional contact, the AO-nut substitutes for the missing lateral cortex. In our hands, it seems to have the same clinical success for these problems as the locking plate.^7^ However, the question is whether the stiffness and strength of the AO-nut augmented construct is mechanically non-inferior to the locking screw.

Our hypothesis is that the AO-nut augmented construct (1) has a stiffness and yield strength that is non-inferior to that of the locking screw construct and (2) results in consolidation of subtrochanteric revision surgery with minimal interface related complications.

## Methods

### Biomechanical testing

In this pilot study a standard 4.5/5.0 mm large-fragment locking compression plate (LCP) (stainless steel, DePuy Synthes, Amersfoort, The Netherlands) and a standard 4.5 mm non-locking dynamic compression plate (DCP) (stainless steel, DePuy Synthes, Amersfoort, The Netherlands) were used as models for the distal part of the 95-degree condylar blade plate. With corresponding 4.5 mm non-locking screws, the 4.5 mm DCP was converted to a fixed-angle construct using AO-nuts. This construct and the locking screw construct were tested mechanically.

For each construct a total of ten screws (n = 10) were tested. Using all holes on each plate without retesting any holes or screws, a total of twenty screws were tested. Per test a single plate-screw construct was created consisting of one 5.0 mm locking screw inserted in one of the ten holes of the 4.5/5.0 mm LCP or a 4.5 mm non-locking screw inserted in one of the ten holes of the 4.5 mm DCP. In the latter, the screw was locked into the holes of the plate by a 4.5 mm AO-nut. To best simulate clinical practice all locking screws were tightened using a torque-limiting screwdriver (4.0 Nm) and the non-locking screws/AO-nut construct were maximally tightened, using a wrench and screwdriver.

**Fig. 2:**
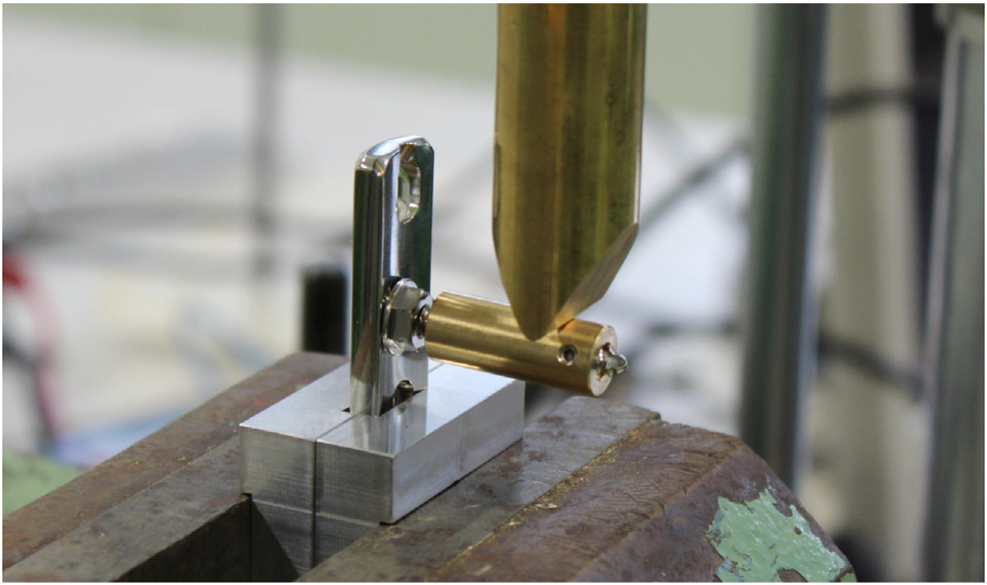
Test setup for the AO-nut augmented construct.

**Fig. 3:**
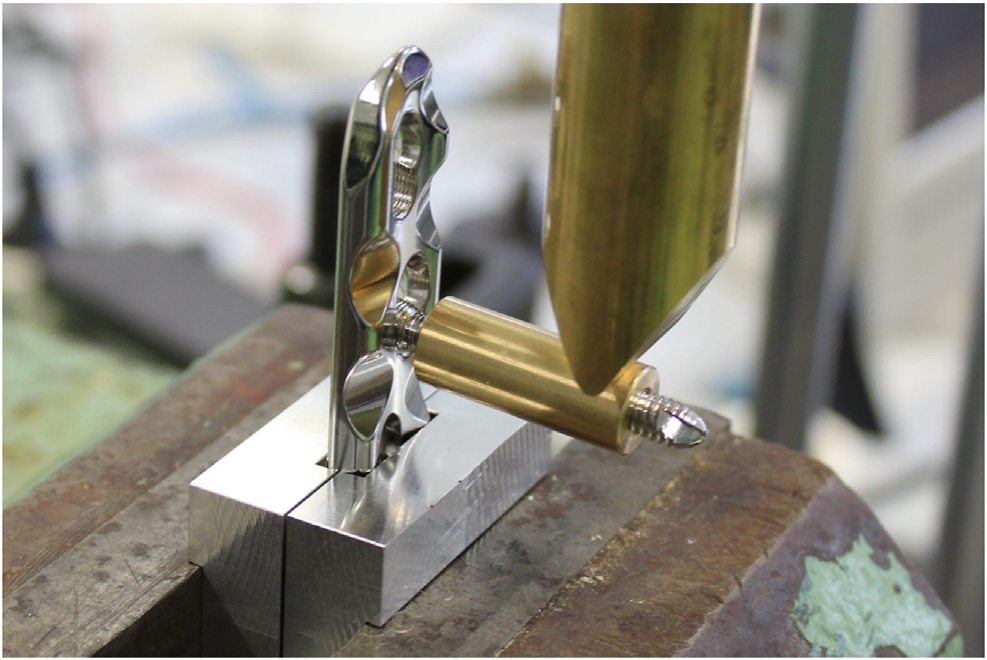
Test setup for the locking compression construct.

**Fig. 4:**
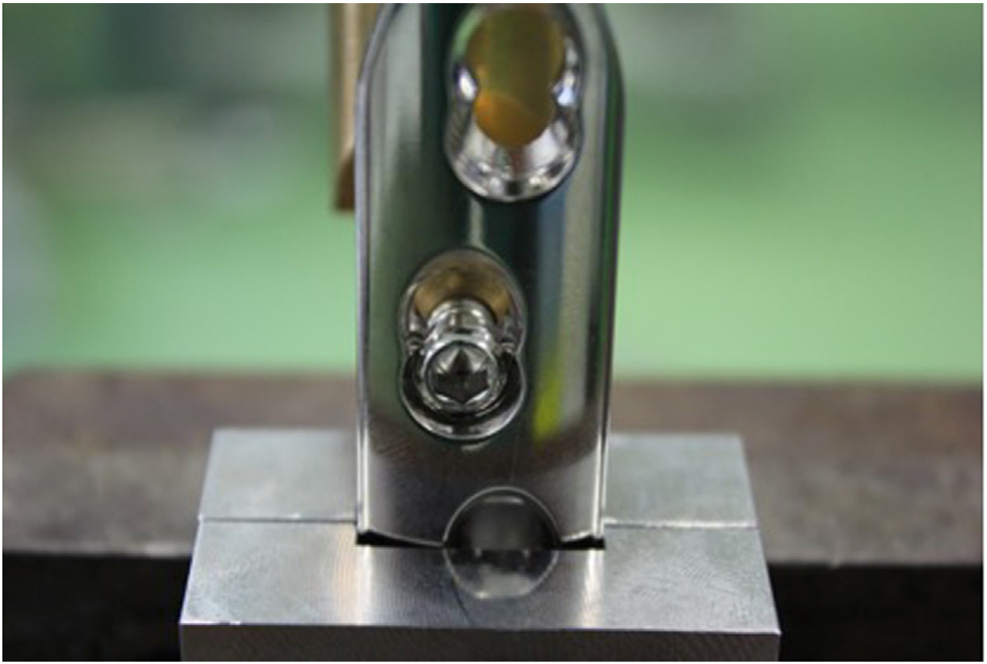
The dynamic compression part of the combi-hole of the LCP was oriented opposite to the loading direction.

The plates were clamped to the testing device. Due to the concave shape of both plates, fixation in the vise would lead to deformation of the plate, possibly affecting the outcome of the experiment. Therefore, two supports were designed for fixation of the LCP and DCP in the vise. A horizontal pin in the support prevented vertical movement of the plates and a rim on the support prevented sliding of the plates in the vise. Thus standardized the distance of 10 mm between the vise and the screw (Fig. 2, Fig. 3). In the test setup the dynamic compression part of the combi-hole of the LCP was oriented opposite to the loading direction, so that during cantilever bending, the screw head was tilted towards the open side of the combi-hole.^8^ (Fig. 4).

A perfectly fitting sleeve was slid over the screw to avoid the bending deformation of the screw in a similar way as a screw that is contained in bone. A distance of 2 mm was taken between the end of the sleeve facing the screw head and the surface of the plate/AO-nut containing the screw.^9^ (Fig. 2, Fig. 3).

To create a bending moment at the locking interface, both screw and sleeve were used as a cantilever. The load was applied by a material-testing machine (Instron) equipped with a 10 kN loadcell. A constant displacement (0.5 mm/min) was applied at a distance of 25 mm from the surface of the plate.^10^ (Fig. 2, Fig. 3).

The screw was loaded until failure occurred. Failure was defined as breakage of the screw or screw-plate construct. During loading, the force and displacement were recorded, with a sample rate of 100 samples/sec. The yield load was determined from the force-displacement data. The point where the load-deformation curve deviated from the initial linear section was estimated by fitting a line to the data until the coefficient of determination R^2^ dropped below 0.996. The measured force at this limit was considered the linear force limit and as such considered as the strength of the construct. This was assumed to be the elastic load limit of the plate-screw construct and the point of irreversible damage. To determine the effect on the outcomes of the assumed value for R,^2^ the calculations were repeated for R^2^ equalling 0.995 and 0.997 using Matlab (R2013B, Natick, MA).

### Clinical series

After the Institutional Review Board waived informed consent, all patient that underwent surgical treatment for subtrochanteric fractures, malunion or non-union at the Academic Medical Center between June 2000 and June 2019 by the senior author (P.K.) underwent chart review. From the medical records, patient demographics, hardware specifications, bone grafting technique and complications were collected.

### Statistical analysis

Statistical analysis was performed with the use of SPSS software (SPSS version 21.0, Chicago, IL). Because of the low number of measurements, normality of the data distribution was not evaluated. In view of the type of test, i.e. mechanical testing of a whole metal construct, normality can be assumed. For statistical testing the unpaired Student’s T-test was used. Statistical significance was defined as p < 0.05.

## Results

### Biomechanical comparison

**Fig. 5:**
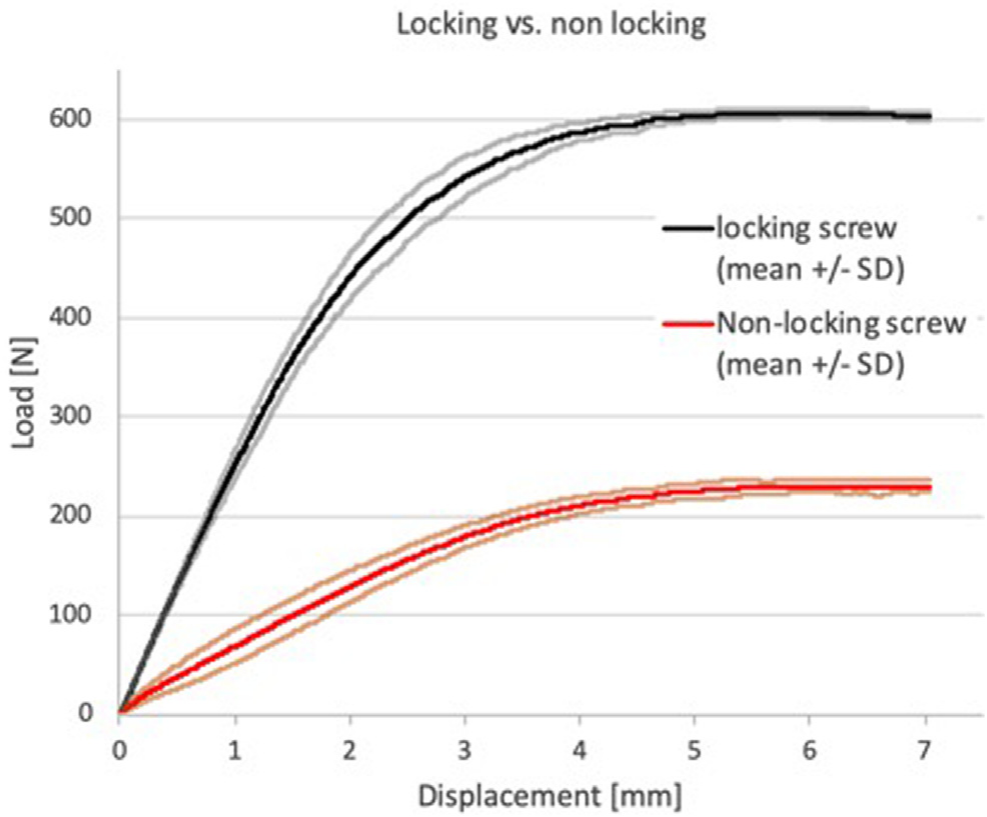
Load-displacement data of the locking- (N = 8) and non-locking (N = 8) plate-screw constructs (mean ± S.D.).

**Fig. 6:**
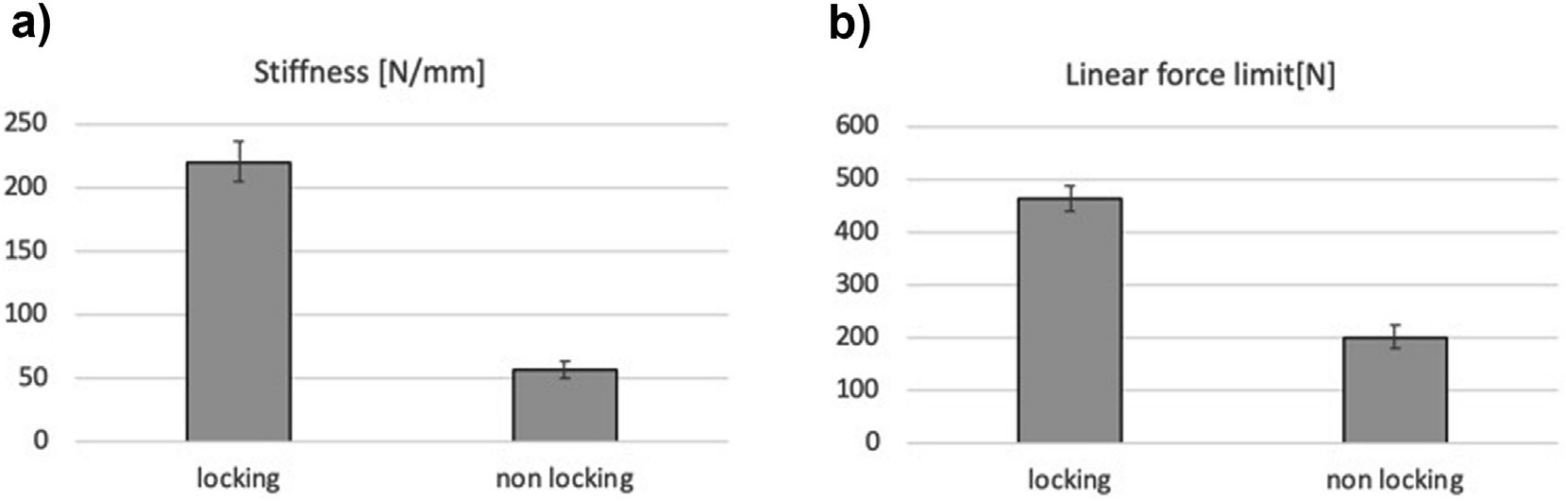
Stiffness (A; N = 8) and linear force limit (B; N = 8) of the locking- and non-locking plate-screw constructs (mean ± S.D.). The differences between locking and non-locking are statistically significant (unpaired Student’s T-test; p < 0.001).

The analysis was performed for eight locking screw-plate constructs and eight non-locking plate-screw constructs for which the average force-displacement data was determined (Fig. 5). Data of four experiments (2/10 locking screws; 2/10 AO-nut augmented constructs) could not be used, due to an error in the registration software. The stiffness of the locking plate-screw construct was about four times higher than that of the AO-nut augmented construct (Fig. 6A). The linear force limit, i.e. the maximal force for which elastic deformation was assumed was about 2.3 times higher for the locking construct than for the non-locking construct (Fig. 6B). The differences between the two constructs were statistically significant (P < 0.001).

Variation of the coefficient of determination to determine the linear force limit affected the mean stiffness by 0,2% and 1.6% for the locking construct and non-locking construct respectively and the mean linear force limit by 0,4% and 1.5% respectively.

Macroscopic investigation after biomechanical testing demonstrated failure at the head screw interface for the locking screws and failure at the screw nut interface in the non-locking screws.

### Clinical experience

Between June 2000 and June 2019 eight patients were treated with before mentioned fixed-angle blade plate construct (Table 1; Fig. 1). In three patients blade plate fixation was indicated for a subtrochanteric fracture, in four patients blade plate fixation was indicated for non-union of a subtrochanteric fracture (one of them was a septic non-union) and one patient underwent a double oblique osteotomy for a malunion. Five out of eight patients healed uneventfully. One patient showed consolidation but died 5 months post-operatively due to unrelated disease. One patient developed a post-operative wound infection requiring multiple incision and drainage procedures. To adequately treat the infection, the patient ultimately underwent AO-nut augmented blade plate revision after which the fracture consolidated. Finally, one patient did initially not heal and underwent revision with a AO-nut augmented blade plate. Although CT scan suggested at least partial bridging, he had ongoing pain in his hip and elected total hip arthroplasty. At the time of hip arthroplasty, the surgeon noticed solid bone healing.

**Table 1:** Demographics and procedural characteristics.

Gender	Age	Indication	Procedure	Bone graft	Outcome
M	58	Infected non-union ^α^	Osteosynthesis	Allograft + ICA	Consolidation, died 5 months post-surgery
F	84	Length discrepancy	Double oblique osteotomy	Allograft + ICA + DBM	Healed
M	77	Infected non-union ^β^	Osteosynthesis	ICA	2x Revision for hardware breakage; long-stem hip prothesis
M	55	Fracture	Osteosynthesis	–	Healed
F	72	Fracture	Osteosynthesis	DBM	Healed
F	53	Non-union	Osteosynthesis	RIA-autograft femur	Healed
M	88	Fracture	Osteosynthesis	–	Healed after blade plate revision for infection ^γ^
F	46	Non-union	Osteosynthesis	ICA	Healed

α Staphylococcus Epidermidis, β Staphylococcus Hominis, γ Escherichia coli ICA = Iliac Crest Autograft, RIA = Reamer-Irrigator-Aspirator, DBM = Demineralised Bone Matrix.

## Discussion

The biomechanical results of this study are that the stiffness and strength of the AO-nut augmented construct is 25%, respectively 43% of that of the locking plate-screw construct. This is not entirely unexpected. It is important to point out that we do not know what stiffness and strength is required in the clinical scenario. Post-operative rehabilitation for these patients always consists of toe-touch (10–15 kg) for at least 6 weeks. From a bone healing perspective, a construct that is too rigid (strain rate lower than 2%) will lead to delayed or no healing.^11^ To strive for maximum stiffness and strength, will also likely lead to failure. To place our biomechanical results in a clinical perspective, the use of AO-nut augmented blade plates resulted in good clinical outcomes. This shows that the stiffness of the AO-nut augmented construct is well beyond the biomechanical threshold. We have also used the AO-nut augmented 95-degree condylar blade plate construct in other locations (femoral shaft, distal femur, proximal tibia, proximal humerus, distal tibia) with similar good clinical results. These are however not part of the current series as we elected to focus on a single area with similar loading in the post-operative period.

Prior to the introduction of the locking plates, other designs were aimed at increasing the holding power of standard screws in osteoporotic bone. Jeffrey Mast developed the Schühli that converted a standard screw into a fixed angle screw.^6^ The Schühli locked the screw to the plate making each screw a fixed angle device. There was a spiked undersurface to increase plate-bone friction and to preserve periosteal vascularity. A limited number of studies have investigated the biomechanical properties of the Schühli.^12^, ^13^, ^14^

Kolodziej et al. were the first to demonstrate that although addition of a Schühli did not significantly increase fixation stability, load and angular deformation at failure were significantly increased for the Schühli augmented plates.^12^ These results were later replicated by Simon et al. in a cadaveric humeral shaft fracture model.^14^ Finally, Jazrawi et al. compared Schühli augmentation to the fixation stability of standard screws and cement-augmented screws in a simulated osteoporotic humeral shaft fracture model.^13^ In this study no statistically significant increase of fixation stability for axial and 4-point bending was found with Schühli-augmentation compared to the cement augmented screws or standard screws.

An alternative to the Schühli is the AO-nut, which is somewhat analogous to the Zespol devices.^12^ A contra-nut on the other side of the plate is tightened to the screw making it a fixed-angle type fixation. Although intuitively the current locking screws seem superior to the Schühli and the AO-nut, no biomechanical comparison studies have been performed.

Clinical reports on the use of Schühlis and the AO-nuts have been favorable.^2^^,^^7^^,^^15^ At our institution the AO-nut has been used in external plate fixators and in plate fixation of humeral, proximal tibia, femoral shaft and distal femur fractures. All patients went on to consolidation and no failures of the AO-nut construct have been observed. From an economic point of view, use of the locking plate (LCP) with corresponding screws is approximately three times as expensive, as compared to the AO-nut augmented blade plate construct (€1000 versus €300, including VAT). Therefore, the AO-nut might be a valuable alternative to the more expensive locking plates in the low-resource setting.

There are some limitations to this investigation. Ex-vivo biomechanical testing is inherently unable to accurately simulate the forces acting on the screw-plate construct as experienced during in-vivo loading. In this study simple bending and shear loading was used to test the stiffness and strength of the screw-plate construct, as they are critical to the locking mechanism. Furthermore, minimal bending of the plate might have occurred during loading. However, as plastic deformation of the screw occurred early in the test (around 2 and 4 mm displacement for the locking and non-locking constructs), this is very unlikely. This study focussed on the limit load of a single screw-plate connection. In the clinical setting multiple screws are used to connect the plate to the bone. Full constructs might have been a better representation of the clinical reality but would have been unable to answer the research question of the present paper. Finally, the presented series of patients, managed with the use of the AO-nut augmented blade plate construct, has the inherent disadvantages of a small retrospective case study.

Despite these shortcomings, this study is of clinical importance as it is the first to biomechanically compare the yield strength of the locking mechanism of an AO-nut augmented 4.5 mm DCP with that of a 5.0 mm LCP. The strength of this biomechanical comparison lies in the fact that it tested the yield strength of both constructs in isolation, where biomechanical properties of these constructs are most commonly tested in cadaveric models. Similar studies on yield strength have been performed for poly-axial locking screws and off-axis insertion of screws.^8^^,^^10^^,^^16^, ^17^, ^18^, ^19^ The benefit of testing the yield strength in isolation is that, despite a limited number of tests, reproducible and statistically significant results can be obtained.

Despite the inferior stiffness and strength of the AO-nut augmented blade plate construct, the clinical series demonstrated the excellent clinical outcome that can be achieved utilizing this technique. Absolute rigid fixation with locking plates has been associated with catastrophic hardware failure.^5^^,^^6^ Based on the recently unified theory of bone healing, fractures and nonunions heal most reliable between an ideal strain of 2–5%.^11^ This is generally obtained by using a construct that is neither to stiff (all locking screws) or too flexible (bridging construct). This is easier said than done as there are no strict guidelines as to what the perfect hardware construction is. In compromised bone, such as in a non-union and/or revision, we always aim for a combination of alignment, compression (use of AO-tensioner devise), hybrid fixation, with ideally a lag screw through the plate. The plate should preserve as much bone stock as possible (leaving a small foot print). To combine “the best of both worlds”, a new design of the 95-degree condylar blade plate should contain combi-holes along the shaft to allow for fixation in locking and non-locking fashion. Until then, the AO-nut augmented blade plate fixation remains a valuable alternative to LCP fixation in subtrochanteric revision surgery.

## Conclusion

Although in our experimental design, the stiffness and strength of the AO-nut augmented construct appeared less than the locking screw, excellent clinical outcomes can be achieved utilizing the AO-nut augmented 95-degree condylar blade plate construct in subtrochanteric fracture and nonunion revision cases.

## Conflict of interest

The Author(s) declare(s) that there is no conflict of interest.
